# Copper Ion Removal by Adsorption Using Fly Ash-Based Geopolymers: Process Optimization Insights from Taguchi and ANOVA Statistical Methods

**DOI:** 10.3390/ma17163992

**Published:** 2024-08-11

**Authors:** Loredana Litu, Gabriela Buema, Giannin Mosoarca, Maria Harja

**Affiliations:** 1 Faculty of Chemical Engineering and Environmental Protection, “Gheorghe Asachi” Technical University of Iasi, 73 Prof. Dr. Docent D. Mangeron Str., 700050 Iasi, Romania; llitu@gmail.com; 2 National Institute of Research and Development for Technical Physics, 47 Mangeron Boulevard, 700050 Iasi, Romania; 3 Faculty of Industrial Chemistry and Environmental Engineering, Politehnica University Timisoara, Bd. V. Parvan Nr. 6, 300223 Timisoara, Romania; giannin.mosoarca@upt.ro

**Keywords:** copper removal, friendly materials, geopolymer, heavy metal removal, process optimization, wastewater treatment

## Abstract

The present study aimed to use geopolymer materials synthesized from different fly ashes, which are promising for the adsorption of copper ions from aqueous solutions. The characterization of fly ashes and prepared adsorbents was performed by energy-dispersive X-ray spectroscopy (EDS) analysis, Brunauer–Emmett–Teller (BET) surface area analysis, and Scanning Electron Microscopy (SEM). Taguchi and ANOVA methods were used to predict the effect of different working parameters on copper ion removal by prepared geopolymers. Based on data obtained by the Taguchi method, it was found that the factor most influencing the adsorption process is the type of adsorbent used, followed by the solution pH, the reaction time, the adsorbent dose, and the initial copper ion concentration. The ANOVA results agree with the Taguchi method. The optimal conditions of the adsorption process were: fly ash C modified by direct activation with 2 M NaOH, at 70 °C for 4 h, solution pH of 5, initial pollutant concentration of 300 mg/L, 40 g/L adsorbent dose, and 120 min of reaction time. Copper ion removal efficiency was determined experimentally under optimal conditions, achieving a value of 99.71%.

## 1. Introduction

Water remediation is recommended for the safety of human health and the environment. The type of material plays an important role in the adsorption process. It was underlined that the cost of adsorbents and the ability of adsorbents to be reused for a number of adsorption/desorption cycles are key parameters for their practical applications in wastewater treatment [1,2]. Apart from many materials, geopolymer materials obtained from wastes are a viable alternative for wastewater treatment. For example, for the removal of cobalt, lead, nickel, and cadmium ions, adsorbents based on pyrophyllite mine waste-based geopolymer [3] and a geopolymer from dolochar ash [4] were proposed. Nayak and co-workers [5] prepared hydroxyapatite synthesized from egg shells and used it for fluoride removal.

Copper is the third most utilized metal in the world [6,7], being used in various industries [8]. These industries must treat their effluent before discharging it [9]. Although copper is an essential element for organisms [10], when consumed in excess, it shows deleterious effects such as irritation of eyes, nose, and mouth, stomachache, lung cancer, and neurotoxicity [11].

Liu and co-workers [6] provided a review focusing on methods used for the removal of copper ions from wastewaters, such as physicochemical techniques (e.g., membrane separation, ion exchange, chemical precipitation, electrochemistry, adsorption) and biological techniques (e.g., biosorption, bioprecipitation, biomineralization).

Currently, the most applied method used for copper ion removal from wastewater is based on adsorption techniques. The method can be applied to industrial wastewater with high copper content. The scientific literature reported copper concentrations in wastewater ranging from approximately 2.5 mg/L to 10,000 mg/L [6]. For example: plating (silver) wastewater 3–900 mg/L, brass mills wastewater 4–888 mg/L, copper mills wastewater 19–800 mg/L, copper sulphate manufacture wastewater 433 mg/L, copper smelting wastewater 200–3500 mg/L [12,13]. Adsorbents that can be used for copper removal are: carbon nanocomposites [14], NTA (nitrilotriacetic acid)-silica gel [15], Azadirachta indica powder [16], sodium hydroxide (NaOH)-treated rice husk [17], modified hematite (α-Fe_2_O_3_) iron oxide-coated sand [18], imidazothiazole Schiff base functionalized silica [19], modified activated carbon [20], alkaline earth metal-based metal-organic frameworks [21], coal gangue [22], and geopolymers [23,24].

By burning coal in a thermal power plant, an industrial solid waste named ‘fly ash’ is obtained [25,26,27]. Though known to have negative impacts on the environment, fly ash, when treated with an alkali reagent, creates a new class of materials called ‘geopolymers’ [28,29,30,31]. The common alkali types, such as NaOH, KOH, NaOH/Na_2_SiO_3_ and KOH/Na_2_SiO_3_ can be used in geopolymer synthesis [32,33,34]. The study of geopolymer materials as potential adsorbents for the treatment of copper-contaminated waters is gaining popularity [35,36]. 

For example, Mužek and co-workers [37] prepared an adsorbent for copper ion removal using a type F fly ash mixed with NaOH and Na_2_SiO_3_ solutions. Four initial concentrations and three temperature values were investigated. The results demonstrate that the prepared geopolymer adsorbent shows excellent ability for copper ion removal. Al-Harahsheh and collaborators [38] synthesized a fly ash-based geopolymer using the alkali activator NaOH. Their data demonstrated that at a pH of 6 and 25 °C, the maximum adsorption capacity of the prepared material was 96.8 mg/g. By increasing the temperature to 45 °C, the maximum adsorption capacity increased to 152 mg/g. The study performed by obtaining glassy ceramic materials and co-workers [33] offered information regarding the preparation of geopolymers using a fly ash collected from Indonesia, treated with four alkali reagents, as adsorbents for copper ion removal. The results show that the treated geopolymers have enhanced adsorption capacities compared to unmodified fly ash. Purbasari and collaborators activated fly ash with 10 N NaOH solution and Na-silicate solution [39]; according to the results, the material is suitable for copper ion removal.

Roviello and co-workers have applied, for the first time, hybrid geopolymeric foams for the removal of different ions, such as Pb^2+^, Cd^2+^, Cu^2+^, and Zn^2+^; the results show that the materials are effective in the adsorption of the targeted ions [40].

Harja and collaborators [41] conducted a study focused on the treatment of copper-contaminated waters by geopolymer materials derived from fly ash. All the developed adsorbents were obtained by treating a locally sourced fly ash with NaOH using different synthesis conditions and synthesis methods at mild temperatures (<100 °C) for a short period of time. The new products have a significant influence on the reduction of the negative impact of fly ash on the environment (related to its storage). In addition, copper-contaminated waters were treated. The study revealed that the prepared adsorbents show good removal efficiencies. The mentioned study was continued with a principal research objective of establishing the best experimental process conditions in order to obtain higher removal efficiency. Thus, to optimize the working parameters used for copper ion removal (i.e., pH, adsorbent dosage, contact time), our research team studied the neuro-evolutionary method, which incorporates neural modeling and genetic algorithm optimization [42].

The scientific literature indicates several optimization methods for the removal of metal ions from wastewater by adsorption, such as the Taguchi approach, Plackett–Burman Design, and Response Surface Methodology (based on three-level full factorial design, Box–Behnken design, central composite design, or Doehlert design) [43,44,45]. The optimization of copper ion removal was investigated by Response surface methodology [44], and the optimization of lead ion removal was investigated by Box-Behnken design [45]. Bayuo and co-workers [43] present in their critical literature review the Response surface optimization and modeling in heavy metal removal from wastewater. The optimal conditions for the copper adsorption process can be established by using the Taguchi method [46,47]. This method is applied to optimize various processes in a wide range of areas, being able to find an optimized design configuration for multifactorial conditions. Compared with other optimization methods, the approach of ranking the controllable factors that influence the analyzed process allows a better visualization of the optimal conditions and requires much less experimental data. The major advantages of the Taguchi method are: (i) keeping the experimental cost to a minimum because a small number of trials are carried out; and (ii) reducing the time of experimental studies and establishing the most effective parameter that influences the process [48,49,50].

The Taguchi method is based on the realization of an orthogonal matrix that distributes the variables in a balanced way, and the experimental results are converted into a signal/noise ratio (S/N), which describes the level of dispersion and the degree of optimization in relation to the desired value [51]. The term ‘signal’ represents the desired value (mean) for the output characteristic, while the term ‘noise’ represents the undesired value (standard deviation) [48,49,52]. For example, if there are six controllable factors at three levels, a fully classical factorial design must use a number of 36, i.e., 729 experiments, to establish optimal conditions that characterize the process. In this case, the Taguchi method uses an L27 orthogonal matrix, which reduces the number of experiments to 27 [49,53].

The study highlights the possibility of using fly ash-based geopolymer materials for copper ion removal and gives detailed information regarding the process optimization insights from Taguchi and ANOVA statistical methods. The Taguchi method was utilized to establish the optimal conditions for the copper ion removal process by adsorption technique [54,55]. The ANOVA analysis (general linear model) was used to determine the contribution of each working parameter on the removal efficiency [56,57]. Five process variables were considered for the present research: type of adsorbent, solution pH, adsorbent dose, initial copper concentration, and reaction time. To the best of our knowledge, there are no data regarding the optimization conditions of copper ion removal using Taguchi and ANOVA methods for geopolymer materials synthesized from two different fly ash sources. 

The following aspects are the strong points that make this study novel for a wide public: (1) the starting material, fly ash, does not involve any costs for purchase; (2) the inexpensive and simple preparation technique (the materials do not require more chemicals and energy); (3) a one-step process for the synthesis of fly-ash-based geopolymers is foreseen, which ensures nearly 100% recovery. 

## 2. Materials and Methods

### 2.1. Materials and Reagents

For fly ash-based geopolymer synthesis, two types of fly ash were used: local Romanian fly ash, Holboca Iasi, (Cen1) and imported Czech Republic fly ash (Cen2). The two types of fly ash were selected for chemical reasons. The Cen1 fly ash is type F (the sum of silicon, aluminum, and iron oxides is over 75%), while Cen2 fly ash is type C, which means it contains more than 7% calcium oxide. NaOH was purchased from Chemical Company, Lasi, Romania (ACS reagent, ≥97.0%, pellets).

### 2.2. Materials Preparation

A number of five adsorbents were synthesized using NaOH. The synthesis conditions are listed in Table 1. The synthesis of CenNa1, CenNa2, and CenNa3 was completed at room temperature (20 °C). The CenNa1 adsorbent was synthesized by treating Cen1 fly ash with 2 M NaOH solution, for 168 h of contact time. The Cen1 to NaOH ratio was 1:3. The CenNa2 adsorbent was synthesized by ultrasound method for 1 h of contact time using a Cen1 to NaOH ratio of 1:3 and a NaOH concentration of 2 M. The CenNa3 was obtained by treating Cen2 fly ash with 2 M NaOH solution by ultrasound method for 1 h of contact time. For CenNa4 and CenNa5 adsorbents, the direct activation method (70 °C) for a contact time of 4 h was used: CenNa4 was prepared by mixing Cen1 with NaOH, 2 M (ratio of 1:3), while the CenNa5 product was synthesized by mixing Cen2 with NaOH, 2 M (ratio of 1:3). After preparation, the adsorbents were kept in closed laboratory bottles.

### 2.3. Materials Characterization

For morphologic and chemical characterization, a Vega Tescan, 3 SBH (Brno, Czech Republic) and QUANTA 3D-AL99/D8229 (FEI, Hillsboro, OR, USA) were used.

The specific surface area BET was determined with Autosorb 1 MP—Adsorption System (Quantachrome Instruments, Boynton Beach, FL, USA).

### 2.4. Copper Ion Adsorption

The copper ion adsorption process was performed using a batch technique at room temperature with stirring. A stock solution of copper sulphate (1000 mg/L) was prepared. The working solutions of 300 mg/L, 500 mg/L, and 700 mg/L were obtained by diluting the stock solution. Distilled water was used in all experiments. For each type of adsorbent, the influence of the solution pH (2–5), adsorbent dose (10–40 mg/L), initial concentration, and reaction time was recorded. Thus, the influence of the type of adsorbent on the adsorption of copper ions was established. The maximum value of pH was selected at 5 to avoid copper ion precipitation. The adsorbents had good stability in the 2–5 pH range, a fact demonstrated in our previous paper [32].

The collected samples after the adsorption process were filtered using filter paper and the filtrate was analyzed spectrophotometrically using a Shimadzu UV-2450 DR UV-Vis spectrophotometer at λ = 390 nm, rubeanic acid.

The removal efficiency was determined by Equation (1) below:

(1) $$R=\left(\frac{C_{0}-C_{f}}{C_{0}}\right)\times 100$$

where $C_{0}$ and $C_{f}$ are the initial and final copper ion concentrations (mg/L).

### 2.5. Optimizing the Adsorption Process

The aim of the optimization studies was to maximize the efficiency of the adsorption process. Therefore, to process experimental data, the calculation ‘larger is better’ (Equation (2)) was considered to determine the $\frac{S}{N}$ ratio [48,58].

(2) $$\frac{S}{N}=-{log}_{10}\left[\frac{1}{n}\sum_{i=1}^{n}{\left(\frac{1}{y_{i}}\right)}^{2}\right]$$

where: *n* is the number of repetitions under the same experimental conditions and $y_{i}$ is the experimental response.

A Taguchi mixed experimental design (18 different experiments—L18) was used to optimize the adsorption process [59]. The effect of five controllable factors on the adsorption efficiency was studied: adsorbent type at 6 levels, pH, adsorbent dose, initial concentration, and contact time at three levels. For a fully classical factorial design, 486 (6 × 34) experiments would have been required.

ANOVA (general linear model) analysis of variance was used to evaluate the results obtained by the Taguchi method and to determine the percentage contribution of each factor on the adsorption efficiency. All mathematical calculations required for the Taguchi method and ANOVA analysis were performed using Minitab 19 statistical software.

## 3. Results

### 3.1. Materials Characterization

Table 2 shows the measured BET surface areas of the modified materials. Furthermore, it was determined that the pore volumes ranged from 0.0233 to 1.35 cm^3^/g, representing an 8.0-fold increase over the initial fly ash material.

The BET investigation shows that the surface area of CenNa1 adsorbent increased 1.29 times the surface area of Cen1 (5.8 m^2^/g). According to Table 2, CenNa1–CenNa5 have higher BET surfaces than Cen1 or Cen2 (7 m^2^/g); the increase can reach up to 10 times.

**Figure 1:**
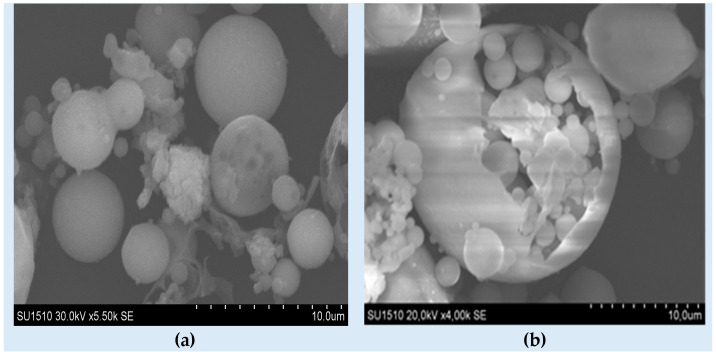

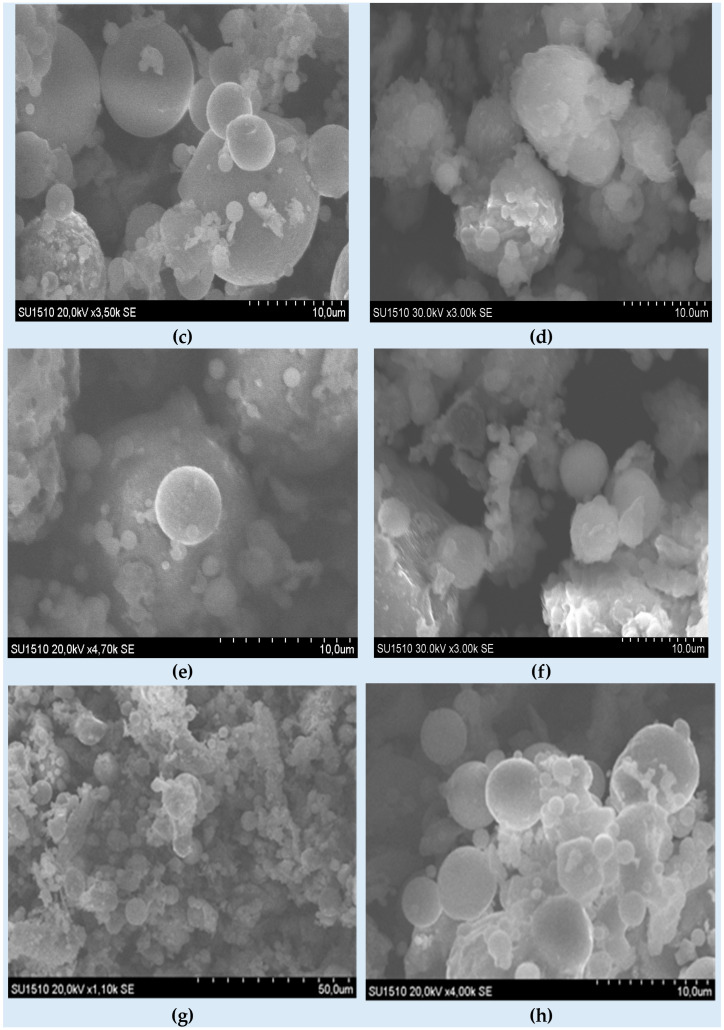
SEM analysis of materials: (**a**) Cen1, (**b**) Cen2, (**c**) CenNa1, (**d**) CenNa2, (**e**) CenNa3, (**f**) CenNa4, (**g**) CenNa5 at 50 µm and (**h**) CenNa5 at 10 µm.

Large modification by alkaline attack is observed in SEM images, Figure 1. On the surface of spherical particles, new crystalline phases are growing; CenNa4 and CenNa5 have the most significant new phases, a fact confirmed by EDS analysis. The EDS analysis was performed to prove that fly ash-based geopolymer materials were successfully synthesized. The data for the local (Cen1) and imported fly ashes (Cen2) are included as well.

The EDS characterization, Table 3, proves that, regardless of the source, both fly ash samples contain O, Na, Mg, Al, Si, K, Ca, Ti, and Fe. Regarding carbon content, a value of 6.72% was found in Cen1, and only 1.25% in Cen2, this depending on burning conditions. Sulfur was not found in source materials. By analyzing the data, it can be noted that Cen2 contains a higher calcium content (6.85%), while Cen1 shows 1.15% calcium. In the synthesized adsorbents, the same components as in the starting material (Cen1 and Cen2) were found, but in different quantities, and the increased sodium content demonstrates the successful modification. Thus, Na content increased for CenNa1, CenNa2, and CenNa4 materials to 3.45%, 2.82%, and 6.71%, respectively, compared to Na content of Cen1 (0.79%) [60]. Regarding the CenNa3 and CenNa5 materials, the Na content was 3.92% and 5.67% compared to Na content of Cen2 (0.603%). 

It can be noted that the lower Na content is present in fly ash-based geopolymer materials prepared using the ultrasound method for 1 h of contact time. The clear increases in geopolymers’ BET surface area and pore volume as compared to the original ashes have further shown the latter material’s potentially improved capacities for copper adsorption. Superior results in terms of adsorption capacity for CenNa4 and CenNa5 can be explained by the advanced degree of modification, wide surface area, and high sodium concentration. 

### 3.2. Taguchi Model and Statistical Analysis

Table 4 reports the controllable factors and their levels used in the Taguchi mixed design. For this study, Cen1 and all five synthesized fly ash-based geopolymer products were considered.

According to the Taguchi design used, 18 different experiments were performed using the L18 orthogonal matrix, and the adsorption efficiency values and S/N ratios corresponding to each iteration were determined (Table 5).

Table 6 shows the S/N ratio for each controllable factor along with the delta values (differences between the highest and lowest mean response values for each factor). The ranking levels of the S/N ratios established based on the delta values are also shown in Table 6. The optimal conditions to obtain the highest adsorption efficiency correspond to the highest value of the S/N ratio for each controllable factor. These values are marked in Table 6 with an ‘*’ symbol.

**Figure 2:**
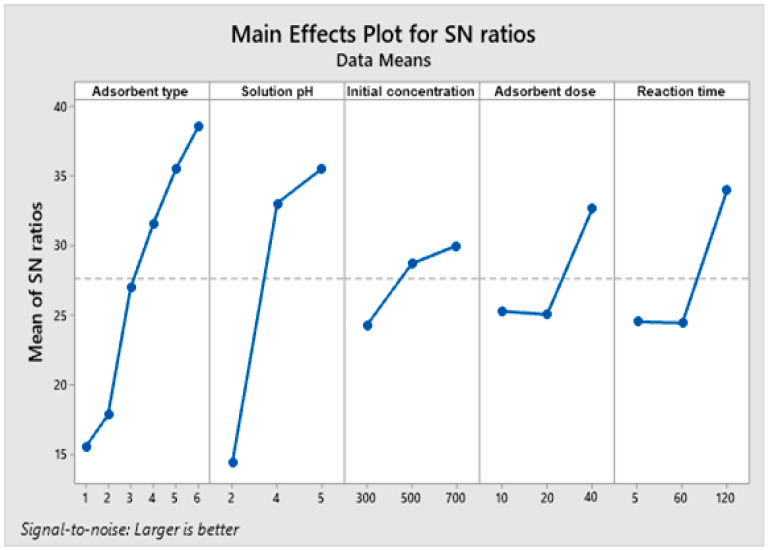
The variations of the S/N ratios for each controllable factor in the pollutant adsorption process on the adsorbent materials.

The variations of the S/N ratios for each controllable factor in the pollutant adsorption process are illustrated in Figure 2. Analyzing the data presented, it can be found that the factor that most influences the adsorption process is the type of adsorbent material used, followed by the solution pH, the reaction time, the adsorbent dose, and the initial concentration of the pollutant.

The correlation of these data with those in Table 1 indicates the optimal conditions of the adsorption process: CenNa5 adsorbent, solution pH of 5, initial concentration of the pollutant of 700 mg/L, 40 g/L adsorbent dose and 120 min. of reaction time. Copper ion removal efficiency was determined experimentally under optimal conditions, obtaining a value of 99.71%. 

**Figure 3:**
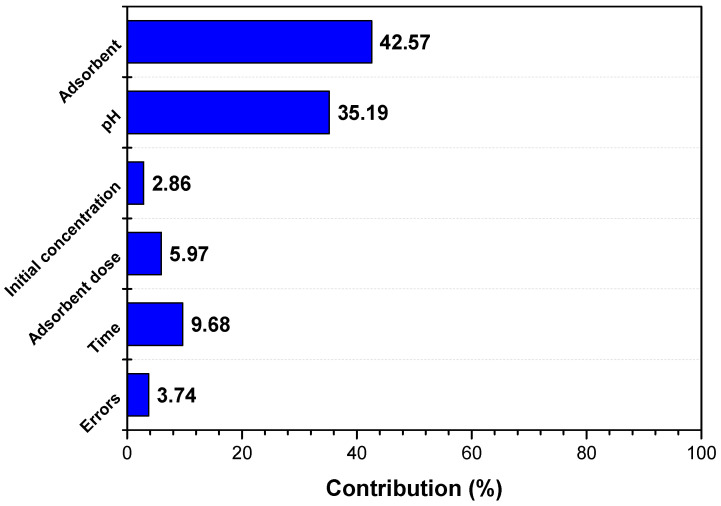
Percentage contribution of each controllable factor on pollutant removal efficiency calculated using general linear model (ANOVA) analysis of variance.

The results of the ANOVA analysis confirm the results obtained by the Taguchi method, indicating the same order of influence of the controllable factors on the adsorption process. The percentage contribution of each controllable factor on pollutant removal efficiency, determined by ANOVA analysis of variance, is shown in Figure 3. Table 6 and Figure 3 do not provide the values of pH or other controllable factors, but the values for the S/N ratio obtained from the Taguchi design analysis, for each individual level.

**Figure 4:**
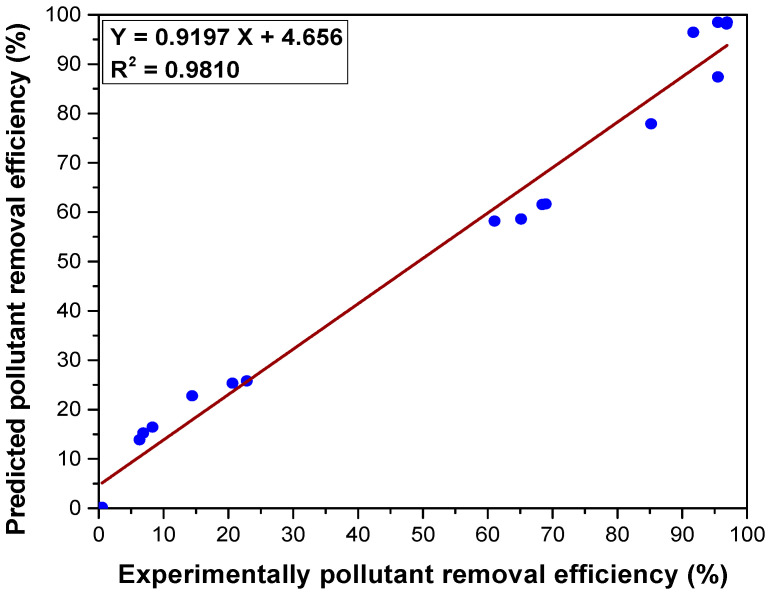
Correlation between predicted and experimentally determined values of pollutant removal efficiency.

To establish the accuracy of the results provided by the Taguchi method, the predicted values of the pollutant removal efficiency and those determined experimentally were correlated (Figure 4). A good correlation between the values was found, with the value of the coefficient of determination R^2^ being very good, indicating good accuracy in the prediction of the optimization method.

A comparison with the existing literature shows that the dominant controllable factor influencing copper ion adsorption depends on the specific adsorbent material used. Svilović and collaborators [47] studied the adsorption of copper ions on fly ash-based geopolymer, modified fly ash-based geopolymer with Pb ions, and zeolite NaX. After Taguchi optimization, it was concluded that the factor with the greatest influence was the copper initial concentration, followed by the adsorbent type and contact time. Another research conducted by Zarandi and collaborators [61] involved the adsorption of copper ions utilizing magnetic nanoparticles attached to activated carbon. Following Taguchi’s approach, it was determined that the most influential controllable factor was the adsorbent dose, followed by pH and copper initial concentration.

**Figure 5:**
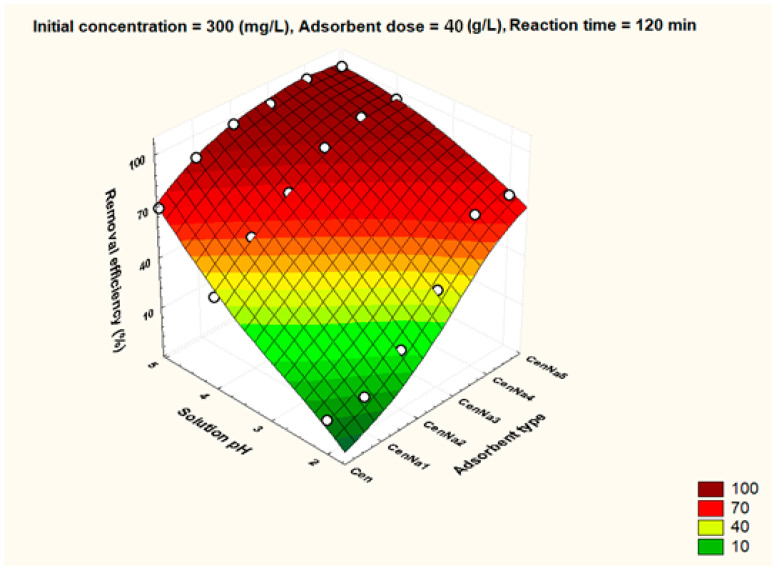
The dependence between the type of adsorbent, solution pH and removal efficiency.

**Figure 6:**
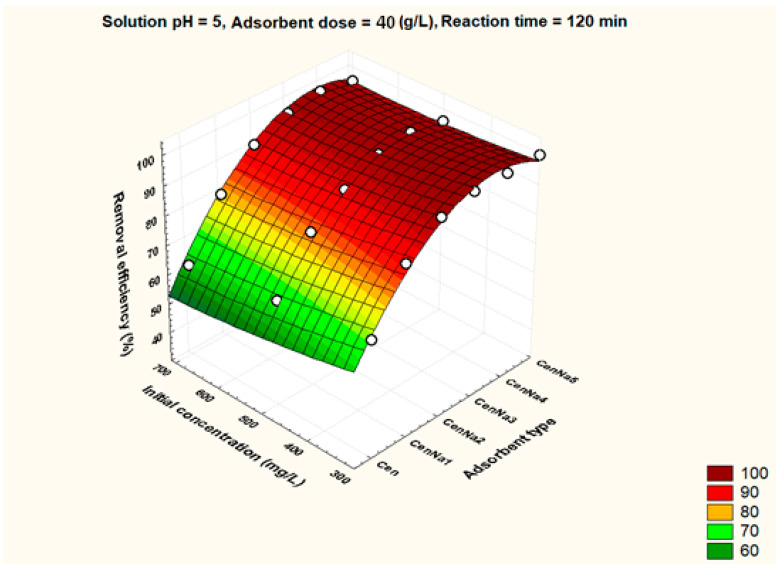
The dependence between type of adsorbent, initial concentration of the pollutant and removal efficiency.

**Figure 7:**
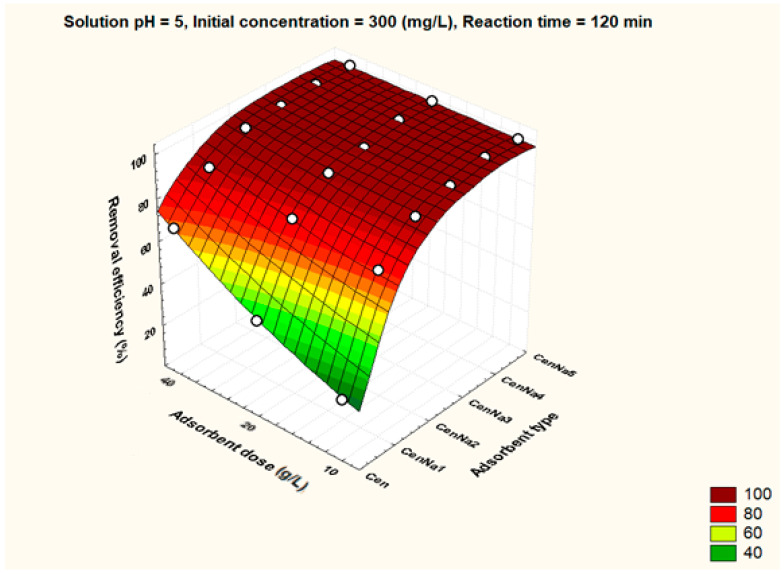
The dependence between type of adsorbent, adsorbent dose and removal efficiency.

**Figure 8:**
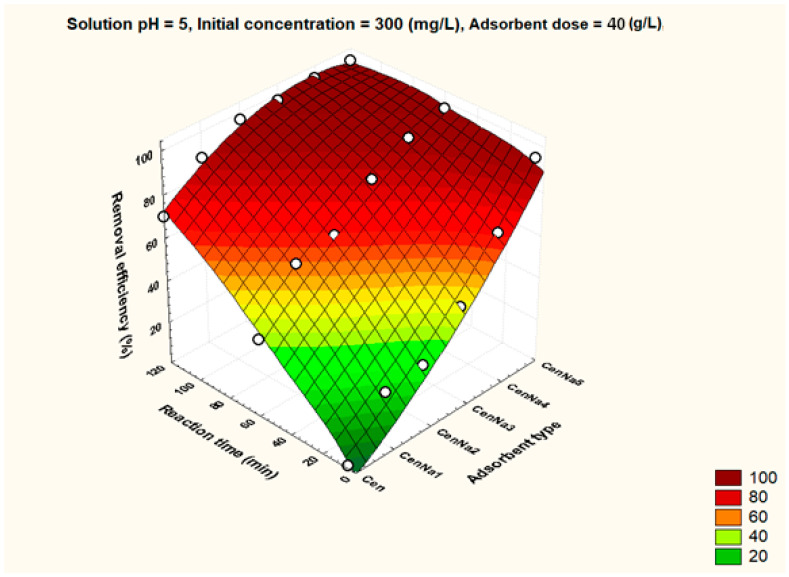
The dependence between adsorbent type, reaction time and removal efficiency.

**Figure 9:**
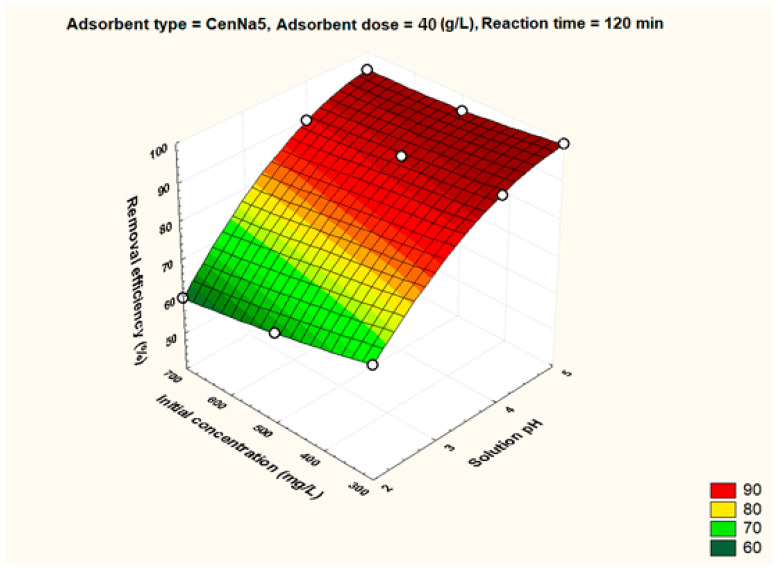
The dependence between solution pH, initial pollutant concentration and removal efficiency for CenNa5 adsorbent.

**Figure 10:**
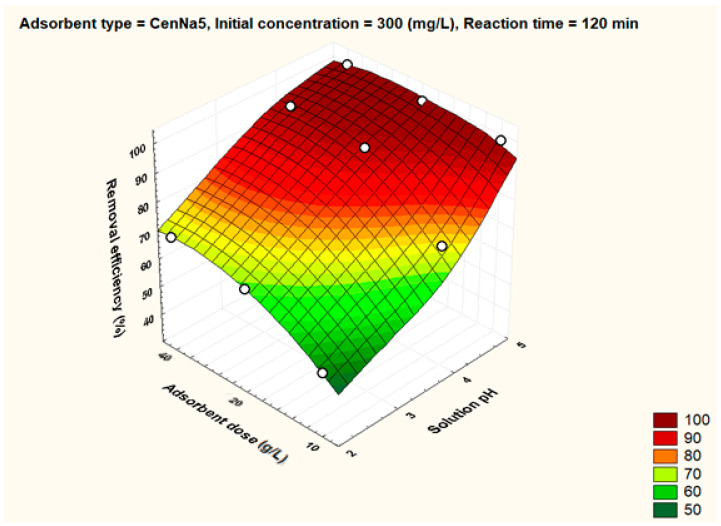
The dependence between solution pH, adsorbent dose and removal efficiency for CenNa5 adsorbent.

**Figure 11:**
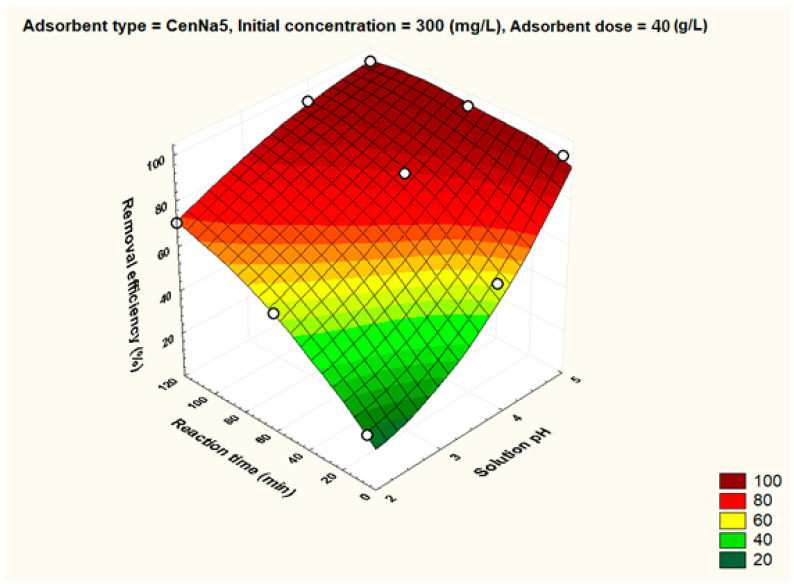
The dependence of solution pH, reaction time and removal efficiency for CenNa5 adsorbent.

**Figure 12:**
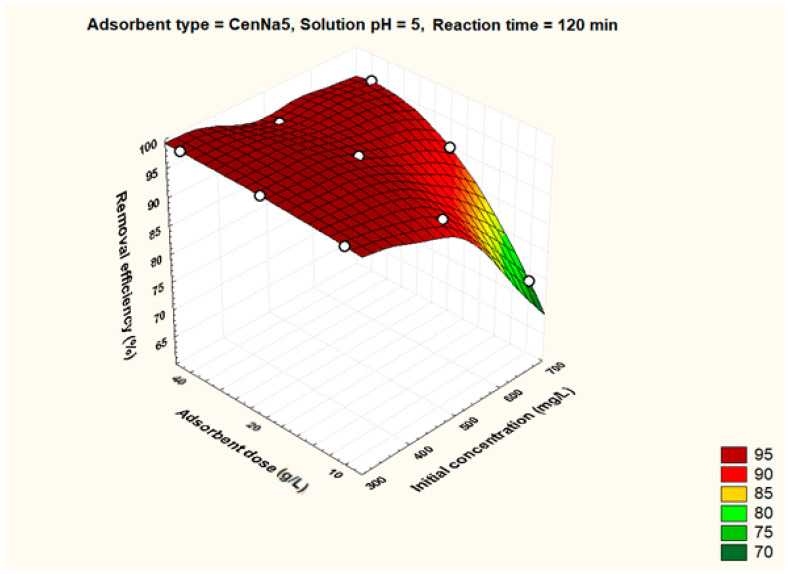
The dependence between initial pollutant concentration, adsorbent dose and removal efficiency for CenNa5 adsorbent.

**Figure 13:**
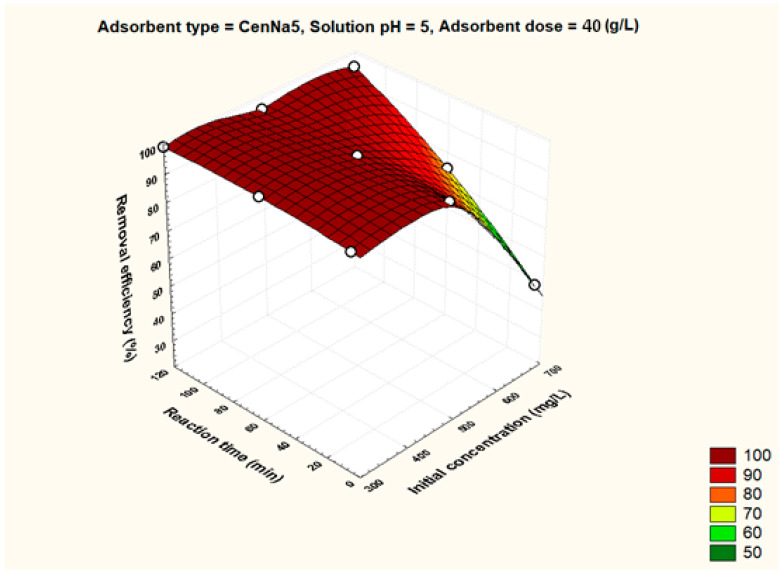
The dependence between initial pollutant concentration, reaction time and removal efficiency for CenNa5 adsorbent.

**Figure 14:**
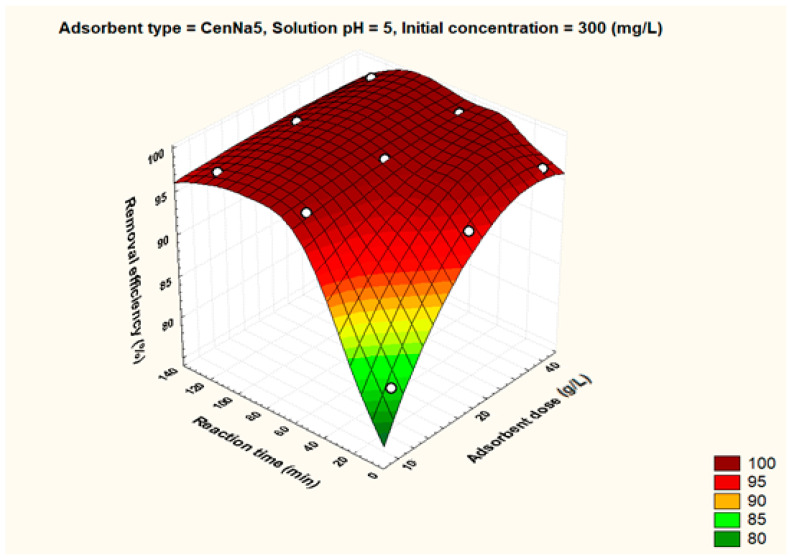
The dependence between adsorbent dose, reaction time and removal efficiency for CenNa5 adsorbent.

To better visualize the influence of the controllable factors on the adsorption efficiency, the dependencies between the main factors that influence the adsorption process and the process efficiency (i.e., optimal conditions established by the Taguchi method—pH 5, 500 mg/L initial copper concentration, and 40 g/L adsorbent dosage) were graphically represented with the STATISTICA 7.1 software using the distance-weighted least squares fitting method. Graphical representations of these dependencies are shown in Figure 5, Figure 6, Figure 7, Figure 8, Figure 9, Figure 10, Figure 11, Figure 12, Figure 13 and Figure 14.

From Figure 5, it can be seen that the lowest removal efficiency is obtained at pH 2, and as the pH increases, the removal efficiency increases. Removal efficiencies close to 100% are obtained at pH 5 using CenNa4 and CenNa5 adsorbents. 

Figure 6 demonstrates that by increasing the concentration of the initial copper solution from 300 mg/L to 700 mg/L, even at pH 5, the adsorption efficiency decreases, which can be explained by the saturation of the adsorption sites.

Under optimal conditions, the adsorbent dose has a positive impact on the adsorption process. Thus, at an adsorbent dose of 40 g/L, the removal efficiency values for unmodified fly ash and for synthesized materials are over 60% (Figure 7).

The reaction time visibly influences the removal efficiency. Operating at optimal reaction time brings significant time and energy savings. Thus, from Figure 8, it was found that for unmodified fly ash and for CenNa1 adsorbent, a removal efficiency above 90% can be reached after 120 min, while the same removal efficiencies can be obtained after a short contact time, i.e., 5–10 min, in the case of CenNa4 and CenNa5.

As observed from the optimization data, the highest removal efficiency values were obtained for CenNa5. Therefore, the influence of each parameter was analyzed for this adsorbent, Figure 9, Figure 10, Figure 11, Figure 12, Figure 13 and Figure 14. However, it can be seen that the differences of CenNa5 when compared to CenNa4 are not significant, so the results can be extrapolated to CenNa4.

Initial pollutant concentrations of 300 mg/L at pH 5 lead to removal efficiencies higher than 95%. The fact that the pH producing the highest efficiencies is closer to neutral pH is beneficial. Although the modified fly ashes are slightly basic (the modification is carried out with NaOH solution), in water treatment plants, neutralization is practiced (mixing acidic and basic waters) so the pH of industrial waters is close to the optimal pH values arising from experimental research.

For CenNa5 at pH 5 and initial concentrations of 300 mg/L, the removal efficiency is above 80% even after 10 min of reaction time.

Experimental data for wastewater with an initial concentration of 300 mg/L revealed removal capacity values of 23.5 mg/g and 5.65 mg/g for the CenNa2 material using adsorbent dosages of 10 mg/L and 40 mg/L, respectively, while for CenNa3 the values obtained were 17.98 mg/g and 5.17 mg/g at the same dosages. Regarding CenNa4 and CenNa5, removal capacity decreased with adsorbent dosage, being 26.9 mg/g for the adsorbent dosage of 10 mg/L, 13 mg/g for the dosage of 20 mg/L, and 6.92 mg/g for the dosage of 40 mg/L. For all studied materials, removal capacities ranged from 6.9 mg/g to 27.25 mg/g.

The synthesized materials have removal efficiency, at the value of pH 5, in accord with the literature; a comparison with other materials is presented in Table 7.

According to Table 7, the material prepared in this study showed good adsorption capacity.

An important aspect that must be treated with special attention is the safe disposal of the loaded adsorbent. A previous recommendation is made by Maiti and co-workers [69]. The authors proposed to use the material after adsorption of heavy metals in cement matrix. The adsorbent materials used are derived from fly ash and therefore have a high SiO_2_ content. The exhausted adsorbent material, resulting from the adsorption process, can be used to obtain vitreous matrices, which have the advantage of fixing the adsorbed heavy metal ions very well, without the danger of them reaching the environment. There are several studies in which some of the authors of this article were involved that certify these statements [70,71].

## 4. Conclusions

This study explores the fly-ash geopolymers prepared from two fly ashes (local and imported types) in mild conditions of temperature as new potential low-cost and environmentally friendly materials for copper ion removal. The performance of synthesized materials was investigated at different operating parameters. To obtain the optimal conditions for the adsorption process, the Taguchi method was used, while the ANOVA analysis (general linear model) was used to determine the contribution of each working parameter on the removal efficiency.

The factor that most influences the adsorption process is the type of adsorbent used, followed by the solution pH, the reaction time, the adsorbent dose, and the initial copper ion concentration. The results of the ANOVA analysis confirm the results obtained by the Taguchi method, indicating the same order of influence of the controllable factors on the adsorption process. A good correlation coefficient between the values was found (R2 = 0.981), which indicates good accuracy for the prediction of the optimization method by Taguchi method.

Newly designed adsorbent, CenNa, can contribute to clean water having a high removal performance of 99.71%. Further, based on the promising results obtained in the present study, we are looking for in-depth research having the objectives: (i) a detailed characterization including other techniques, (ii) isotherms/kinetics/thermodynamics evaluation, (iii) characterization of the loaded material for the suggestion of an adsorption mechanism, (iv) regeneration for multiple cycles using various desorption agents.

For an industrial plant, the technological parameters and especially the controllable ones are the most important. The management and regulation of these parameters are essential for the proper development of the industrial process, while the pollutant retention mechanism became secondary. That is the reason why the optimization methods, used in industrial practice, focus on controllable parameters.

The adsorbents containing heavy metal ions can be easily regenerated, after which, due to the predominant mineral part, they can be used in the cement industry, or for obtaining glassy ceramic materials. Future studies will be focused on these aspects as well.

Therefore, the study can draw the attention of researchers and the removal of other types of pollutants from wastewaters can be investigated by using the findings and fly ash-based geopolymer materials synthesized by our proposed methods.

## Figures and Tables

**Table 1:** Synthesis conditions.

Initial Materials	Adsorbent	Method	Cen:NaOH Ratio	Temperature, °C	NaOH, M	Contact Time, h
Cen1	CenNa1	Direct activation	1:3	20	2	168
Cen1	CenNa2	Ultrasound	1:3	20	2	1
Cen2	CenNa3	Ultrasound	1:3	20	2	1
Cen1	CenNa4	Direct activation	1:3	70	2	4
Cen2	CenNa5	Direct activation	1:3	70	2	4

**Table 2:** Surface area for synthesized materials.

Adsorbent	Cen1	Cen2	CenNa1	CenNa2	CenNa3	CenNa4	CenNa5
S_BET_, m^2^/g	5.8	7	7.5	21.5	15.7	41.1	85.4
V_pori_ 10^−3^, cm^3^/g	24	23.3	31	89.9	67.5	125.5	135

**Table 3:** Elemental composition of raw and synthesized materials.

Adsorbent	O	Na	Mg	Al	Si	K	Ca	Ti	Fe
Cen1	34.6	0.79	0.6	18.09	32.81	1.25	1.15	0.44	2.15
Cen2	36.23	0.603	0.71	18.23	31.72	0.95	6.85	0.03	3.33
CenNa1	33.85	3.45	0.93	18.33	28.89	0.66	1.44	0.91	4.07
CenNa2	40.87	2.82	0.63	10.58	32.73	0.31	1.33	0.23	0.84
CenNa3	43.05	3.92	0.83	11.06	34.44	0.46	3,33	0.38	1.53
CenNa4	45.31	6.71	1.15	12.79	31.61	0.12	0.60	0.31	1.43
CenNa5	47.78	5.67	0.47	12.14	31.09	0.12	1.10	0.66	0.96

**Table 4:** The controllable factors and their levels used in the Taguchi mixed design.

Factor	Level 1	Level 2	Level 3	Level 4	Level 5	Level 6
Adsorbent	Cen1	CenNa1	CenNa2	CenNa3	CenNa4	CenNa5
Solution pH	2	4	5			
Initial conc. (mg/L)	300	500	700			
Adsorbent dose (g/L)	10	20	40			
Reaction time (min)	5	60	120			

**Table 5:** Experimental results obtained for pollutant removal efficiency using Taguchi L18 orthogonal matrix.

Runs	Adsorbent	Solution pH	Initial Concentration, mg/L	Adsorbent Dose, g/L	Reaction Time, min.	Removal Efficiency, %	S/N Ratio
1	Cen1	2	300	10	5	0.5	−6.02
2	Cen1	4	500	20	60	6.86	16.72
3	Cen1	5	700	40	120	61.06	35.71
4	CenNa1	2	300	20	60	0.5	−6.02
5	CenNa1	4	500	40	120	65.15	36.27
6	CenNa1	5	700	10	5	14.4	23.16
7	CenNa2	2	500	10	120	6.29	15.97
8	CenNa2	4	700	20	5	20.63	26.29
9	CenNa2	5	300	40	60	85.21	38.60
10	CenNa3	2	700	40	60	8.28	18.36
11	CenNa3	4	300	10	120	95.52	39.60
12	CenNa3	5	500	20	5	68.43	36.70
13	CenNa4	2	500	40	5	22.86	27.18
14	CenNa4	4	700	10	60	95.52	39.60
15	CenNa4	5	300	20	120	96.92	39.72
16	CenNa5	2	700	20	120	68.95	36.77
17	CenNa5	4	300	40	5	96.85	39.72
18	CenNa5	5	500	10	60	91.72	39.24

**Table 6:** Response table for S/N ratio (‘larger is better’ option).

Level	Adsorbent	Solution pH	Initial Concentration	Adsorbent dose	Reaction Time
1	15.47	14.37	24.27	25.26	24.51
2	17.81	33.04	28.69	25.03	24.42
3	26.96	35.53 *	29.98 *	32.64 *	34.01 *
4	31.56				
5	35.50				
6	38.58 *				
Delta	23.11	19.15	3.71	6.61	8.59
Ranking level	1	2	5	4	3

**Table 7:** Comparison of removal efficiency of various adsorbents for Cu^2+^.

Adsorbent	Removal Efficiency, %	References
Synthetic mayenite	94.0, after 3 min	[62]
Polish peats	66%, initial concentration of 1000 mg/L	[63]
Crown-ether functionalized graphene oxide	82%, initial concentration of 250 mg/L	[64]
Zeolite NaX	59.37, adsorbent dosage 10 g/L, initial concentration 756 mg/L	[65]
Carbonized zeolite/chitosan composite	85.3, adsorbent dosage 20 g/L, initial concentration 25 mg/L	[66]
Hydroxyapatite	94.62, initial concentration 80 mg/L	[67]
Cement-Based Absorbent Incorporating Fly Ash	98.07, adsorbent dosage 80 g/L, initial concentration 55 mg/L	[36]
MgO nano-adsorbent	99.7, adsorbent dosage 40 g/L, initial concentration 200 mg/L	[68]
Treated fly ash	99.7	This work

## Data Availability

The data presented in this study are available on request from the corresponding authors.

## References

[1] Biswal A.K., Panda L., Chakraborty S., Pradhan S.K., Dash M.R., Misra P.K. (2023). Production of a nascent cellulosic material from vegetable waste: Synthesis, characterization, functional properties, and its potency for a cationic dye removal. Int. J. Biol. Macromol..

[2] Biswal A.K., Sahoo M., Suna P.K., Panda L., Lenka C., Misra P.K. (2022). Exploring the adsorption efficiency of a novel cellulosic material for removal of food dye from water. J. Mol. Liq..

[3] Panda L., Rath S.S., Rao D.S., Nayak B.B., Das B., Misra P.K. (2018). Thorough understanding of the kinetics and mechanism of heavy metal adsorption onto a pyrophyllite mine waste based geopolymer. J. Mol. Liq..

[4] Panda L., Jena S.K., Rath S.S., Misra P.K. (2020). Heavy metal removal from water by adsorption using a low-cost geopolymer. Environ. Sci. Pollut. Res..

[5] Nayak B., Samant A., Patel R., Misra P.M. (2017). Comprehensive Understanding of the Kinetics and Mechanism of Fluoride Removal over a Potent Nanocrystalline Hydroxyapatite Surface. ACS Omega.

[6] Liu Y., Wang H., Cui Y., Chen N. (2023). Removal of Copper Ions from Wastewater: A Review. Int. J. Env. Res. Public Health.

[7] Sočo E., Domoń A., Papciak D., Michel M.M., Pająk D., Cieniek B., Azizi M. (2023). Characteristics of Adsorption/Desorption Process on Dolomite Adsorbent in the Copper(II) Removal from Aqueous Solutions. Materials.

[8] Wu C., Low K.H., Vonika Ka-Man Au V.K.M. (2023). Efficient removal and sensing of copper(II) ions by alkaline earth metal-based metal–organic frameworks. J. Solid State Chem..

[9] Upadhyay U., Sireesha S., Gupta S., Sreedhar I., Anitha K.L. (2023). Freeze v/s air-dried alginate-pectin gel beads modified with sodium dodecyl sulphate for enhanced removal of copper ions. Carbohydr. Polym. Part A.

[10] Gupta N.K. (2023). Adsorptive removal of Cu(II) by fly ash based geopolymer material. Mater. Today Proc..

[11] Danesh N., Ghorbani M., Marjani A. (2021). Separation of copper ions by nanocomposites using adsorption process. Sci. Rep..

[12] Dib A., Makhloufi L. (2004). Cementation treatment of copper in wastewater: Mass transfer in a fixed bed of iron spheres. Chem. Eng. Process..

[13] Liang Q., Jiang L., Zheng J., Duan N. (2024). Determination of High Concentration Copper Ions Based on Ultraviolet—Visible Spectroscopy Combined with Partial Least Squares Regression Analysis. Processes.

[14] Dichiara A.B., Webber M.R., Gorman W.R., Rogers R.E. (2015). Removal of Copper Ions from Aqueous Solutions via Adsorption on Carbon Nanocomposites. ACS Appl. Mater. Interfaces.

[15] Li Y., He J., Zhang K., Liu T., Hu Y., Chen X., Wang C., Huang X., Kong L., Liu J. (2019). Super rapid removal of copper, cadmium and lead ions from water by NTA-silica gel. RCS Adv..

[16] Al Moharbi S.S., Devi M.G., Sangeetha B.M., Jahan S. (2020). Studies on the removal of copper ions from industrial effluent by Azadirachta indica powder. Appl. Water Sci..

[17] Zafar S., Khan M.I., Lashari M.H., Khraisheh M., Almomani F., Mirza M.L., Khalid N. (2020). Removal of copper ions from aqueous solution using NaOH-treated rice husk. Emergent Mater..

[18] Khan J., Lin S., Nizeyimana J.C., Wu Y., Wang Q., Liu X. (2021). Removal of copper ions from wastewater via adsorption on modified hematite (α-Fe_2_O_3_) iron oxide coated sand. J. Clean. Prod..

[19] Cherif A., Alzahrani A.Y.A., Hammoudan I., Saddik R., Tighadouini S. (2023). Synthesis of imidazothiazole Schiff base functionalized silica as an adsorbent for efficient and selective removal of Cu(II) from wastewater: A combined experimental and theoretical investigation. Mater. Today Sustain..

[20] Chen W.S., Chen Y.C., Lee C.H. (2022). Modified Activated Carbon for Copper Ion Removal from Aqueous Solution. Processes.

[21] Wu T., Chen X., Zhang H., Zhao M., Huang L., Yan J., Su M., Liu X. (2023). MoS_2_-encapsulated nitrogen-doped carbon bowls for highly efficient and selective removal of copper ions from wastewater. Sep. Purif. Technol..

[22] Liang Z., Gao Q., Wu Z., Gao H. (2022). Removal and kinetics of cadmium and copper ion adsorption in aqueous solution by zeolite NaX synthesized from coal gangue. Environ. Sci. Pollut. Res..

[23] Yıldırım A.C., Toda K., Saito T. (2024). Determination of the sorption mechanisms of sodium-alkalinized metakaolin-based geopolymers. Appl. Clay Sci..

[24] Elgarahy A.M., Maged A., Eloffy M.G., Zahran M., Kharbish S., Elwakeel K.Z., Bhatnagar A. (2023). Geopolymers as sustainable eco-friendly materials: Classification, synthesis routes, and applications in wastewater treatment. Sep. Purif. Technol..

[25] Ahmed Z.T., Hand D.W., Watkins M.K., Sutter L.L. (2014). Combined Adsorption Isotherms for Measuring the Adsorption Capacity of Fly Ash in Concrete. ACS Sust. Chem. Eng..

[26] Khatib K., Lahmyed L., El Azhari M. (2022). Synthesis, Characterization, and Application of Geopolymer/TiO_2_ Nanoparticles Composite for Efficient Removal of Cu(II) and Cd(II) Ions from Aqueous Media. Minerals.

[27] Küçük İ., Üstündağ P. (2024). Adsorption Performance of Acidic Modified Fly Ash: Box–Behnken design. J. Turkish Chem. Soc. Sec. Chem..

[28] Davidovits J. (2008). Geopolymer. Chemistry and Applications.

[29] Cong P., Cheng Y. (2021). Advances in geopolymer materials: A comprehensive review. J. Traffic Transport. Eng..

[30] Prabhakar A.K., Mohan B.C., Tai M.H., Yao Z., Teo S.L.M., Wang C.H. (2023). Green, Non-Toxic and Efficient Adsorbent from Hazardous Ash Waste for the Recovery of Valuable Metals and Heavy Metal Removal from Waste Streams. Chemosphere.

[31] Su Q., Ye Q., Deng L., He Y., Cui X. (2020). Prepared Self-Growth Supported Copper Catalyst by Recovering Cu(II) from Wastewater Using Geopolymer Microspheres. J. Clean. Prod..

[32] Buema G., Trifas L.M., Harja M. (2021). Removal of toxic copper ion from aqueous media by adsorption on fly ash-derived zeolites: Kinetic and equilibrium studies. Polymers.

[33] Darmayanti L., Kadja G.T.M., Notodarmojo S., Damanhuri E., Mukti R.R. (2019). Structural alteration within fly ash-based geopolymers governing the adsorption of Cu^2+^ from aqueous environment: Effect of alkali activation. J. Hazard. Mater..

[34] Quddus S., Saha M., Hasanuzzaman M., Sharmin N., Bashar M.S. (2024). Low energy synthesis of crystalline mesoporous aluminosilicate consisting of Na-P1 zeolite derived from coal fly ash. Clean. Mater..

[35] Jin H., Liu Y., Wang C., Lei X., Guo M., Cheng F., Zhang M. (2018). Two-step modification towards enhancing the adsorption capacity of fly ash for both inorganic Cu(II) and organic methylene blue from aqueous solution. Environ. Sci. Pollut. Res..

[36] Cai J., Hao M., Zhang R., Xu G., Tian Q., Zhang J. (2023). Removal of Cu^2+^ from Aqueous Solution by Cement-Based Absorbent Incorporating Fly Ash. Water Air Soil Pollut..

[37] Mužek M.N., Svilović S., Zelić J. (2014). Fly ash-based geopolymeric adsorbent for copper ion removal from wastewater. Desalin. Water Treat..

[38] Al-Harahsheh M.S., Al Zboon K., Al-Makhadmeh L., Hararah M., Mahasneh M. (2015). Fly ash based geopolymer for heavy metal removal: A case study on copper removal. J. Env. Chem. Eng..

[39] Purbasari A., Ariyanti D., Sumardiono S. (2020). Preparation and application of fly ash-based geopolymer for heavy metal removal. AIP Conf. Proc..

[40] Roviello G., Chianese E., Ferone C., Ricciotti L., Roviello V., Cioffi R., Tarallo O. (2019). Hybrid Geopolymeric Foams for the Removal of Metallic Ions from Aqueous Waste Solutions. Materials.

[41] Harja M., Buema G., Sutiman D.M., Munteanu C., Bucur D. (2012). Low cost adsorbents obtained from ash for copper removal. Korean J. Chem. Eng..

[42] Curteanu S., Buema G., Piuleac C.G., Sutiman D.M., Harja M. (2014). Neuro-evolutionary optimization methodology applied to the synthesis process of ash based adsorbents. J. Ind. Eng. Chem..

[43] Bayuo J., Rwiza M., Mtei K. (2022). Response surface optimization and modeling in heavy metal removal from wastewater—A critical review. Environ. Monit. Assess..

[44] Maiti S., Prasad B., Minocha A.K. (2020). Optimization of copper removal from wastewater by fly ash using central composite design of Response surface methodology. SN Appl. Sci..

[45] Maiti S., Prasad B., Minocha A.K. (2022). Optimization of Pb(II) removal from aqueous solutions by fly ash using box-behnken design. Environ. Eng. Manag. J..

[46] Pundir R., Chary G.H.V.C., Dastidar M.G. (2018). Application of Taguchi method for optimizing the process parameters for the removal of copper and nickel by growing *Aspergillus* sp. Water Resourc. Ind..

[47] Svilović S., Mužek M.N., Nuić I., Vučenović P. (2019). Taguchi design of optimum process parameters for sorption of copper ions using different sorbents. Water Sci. Technol..

[48] Fernández-López J.A., Angosto J.M., Roca M.J., Miñarro M.D. (2018). Taguchi design-based enhancement of heavy metals bioremoval by agroindustrial waste biomass from artichoke. Sci. Total Environ..

[49] Mosoarca G., Vancea C., Popa S., Boran S. (2021). Bathurst Burr (*Xanthium spinosum*) Powder—A New Natural Effective Adsorbent for Crystal Violet Dye Removal from Synthetic Wastewaters. Materials.

[50] Torisaki M., Shimoda M., Al Ali M. (2023). Shape optimization method for strength design problem of microstructures in a multiscale structure. Int. J. Numer. Methods Eng..

[51] Pastarnokienė L., Potapov E., Makuška R., Kochanė T. (2024). Optimization of microencapsulation of polyaspartic acid ester into UV curable epoxy-acrylate resin using Taguchi method of experimental design. J. Appl. Polym. Sci..

[52] Madan S.S., Wasewar K.L. (2017). Optimization for benzeneacetic acid removal from aqueous solution using CaO_2_ nanoparticles based on Taguchi method. J. Appl. Res. Technol..

[53] Razmi B., Ghasemi-Fasaei R. (2018). Investigation of Taguchi optimization, equilibrium isotherms, and kinetic modeling for phosphorus adsorption onto natural zeolite of clinoptilolite type. Adsorp. Sci. Technol..

[54] Korake S.R., Jadhao P.D. (2020). Investigation of Taguchi optimization, equilibrium isotherms, and kinetic modeling for cadmium adsorption onto deposited silt. Heliyon.

[55] Shojaei S., Shojaei S., Band S.S., Farizhandi A.A.K., Ghoroqi M., Mosavi A. (2021). Application of Taguchi method and response surface methodology into the removal of malachite green and auramine-O by NaX nanozeolites. Sci. Rep..

[56] Santra D., Joarder R., Sarkar M. (2014). Taguchi design and equilibrium modeling for fluoride adsorption on cerium loaded cellulose nanocomposite bead. Carbohydr. Polym..

[57] Maazinejad B., Mohammadnia O., Ali G.A.M., Makhlouf S.H., Nadagouda M.N., Sillanpää M., Asiri A.M., Agarwal S., Gupta V.K., Sadegh H. (2020). Taguchi L9 (34) orthogonal array study based on methylene blue removal by single-walled carbon nanotubes-amine: Adsorption optimization using the experimental design method, kinetics, equilibrium and thermodynamics. J. Mol. Liq..

[58] Zolgharnein J., Rastgordani M. (2018). Optimization of simultaneous removal of binary mixture of indigo carmine and methyl orange dyes by cobalt hydroxide nano-particles through Taguchi method. J. Mol. Liq..

[59] Baha A.A., Tabit K., Idouhli R., Khadiri M.E., Dikici B., Abouelfida A. (2024). Effect of Liquor/(Si/Al) Ratio on Zeolite Synthesis from Fumed Silica and Coal Fly Ash Using the Taguchi Approach. Chem. Afr..

[60] Harja M., Caftanachi M., Fanache M., Ciobanu G. (2023). Fly ash waste for obtaining building materials with improved durability. Sci. Study Res. Chem. Chem. Eng. Biotechnol. Food Ind..

[61] Zarandi M.J.E., Sohrabi M.R., Khosravi M., Mansouriieh N., Davallo M., Khosravan A. (2016). Optimizing Cu(II) removal from aqueous solution by magnetic nanoparticles immobilized on activated carbon using Taguchi method. Water Sci. Technol..

[62] Eisinas A., Ruginyte K., Baltakys K., Demcak S., Dambrauskas T., Balintova M., Stevulova N. (2020). Cu^2+^ ion adsorption by synthetic mayenite and its thermal stability. Ceram. Int..

[63] Charazińska S., Lochyński P., Burszta-Adamiak E. (2021). Removal of heavy metal ions form acidic electrolyte for stainless steel electropolishing via adsorption using Polish peats. J. Water Proc. Eng..

[64] Petrescu S., Avramescu S., Musuc A.M., Neatu F., Florea M., Ionita P. (2020). Crown-ether functionalized graphene oxide for metal ions sequestration. Mat. Res. Bull..

[65] Bašić A., Penga Ž., Penga J., Kuzmanić N., Svilović S. (2023). Zeolite NaX mass and propeller agitator speed impact on copper ions sorption. Processes.

[66] Hidayat E., Yoshino T., Yonemura S., Mitoma Y., Harada H. (2023). A carbonized zeolite/chitosan composite as an adsorbent for copper(II) and chromium(VI) removal from water. Materials.

[67] Mateiuc A.M., Ciobanu G., Luca C., Luca F.A. (2017). Copper(II) adsorption onto hydroxyapatite. Env. Eng. Manag. J..

[68] Ismail M., Jobara A., Bekouche H., Abd Allateef M., Ben Aissa M.A., Modwi A. (2022). Impact of Cu Ions removal onto MgO nanostructures: Adsorption capacity and mechanism. J. Mater. Sci. Mater. Electron..

[69] Maiti S., Malik J., Prasad B., Minocha A.K. (2023). Solidification/stabilisation of Pb(II) and Cu(II) containing wastewater in cement matrix. Environ. Technol..

[70] Lazau I., Vancea C., Mosoarca G. (2013). New vitreous matrix for the lead wastes immobilization. Rom. J. Mat..

[71] Vancea C., Mosoarca G., Negrea A., Latia A., Jurca R.M. (2016). New glass-ceramic matrix for the chromium wastes immobilization. Rom. J. Mat..

